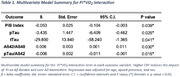# Cardiorespiratory Fitness Modifies the Association Between Cerebral Pulsatility on Amyloid Burden and Core CSF Biomarkers of Alzheimer’s Disease

**DOI:** 10.1002/alz.090733

**Published:** 2025-01-09

**Authors:** Brianne M. Breidenbach, Talia L. Brach, Matthew P Glittenberg, Adam J Paulsen, Leah E Symanski, Tarun Naren, Ira Driscoll, Sarah R Lose, Catherine L. Gallagher, Sterling C. Johnson, Sanjay Asthana, Tobey J. Betthauser, Leonardo A. Rivera‐Rivera, Bruce P Hermann, Mark A. Sager, Kaj Blennow, Henrik Zetterberg, Cynthia M. Carlsson, Gwendlyn Kollmorgen, Clara Quijano‐Rubio, Dane B. Cook, Oliver Wieben, Ozioma C Okonkwo

**Affiliations:** ^1^ Wisconsin Alzheimer's Disease Research Center, School of Medicine and Public Health, University of Wisconsin‐Madison, Madison, WI USA; ^2^ Wisconsin Alzheimer's Institute, Madison, WI USA; ^3^ University of Wisconsin‐Madison, School of Medicine and Public Health, Madison, WI USA; ^4^ Geriatric Research Education and Clinical Center William S. Middleton VA Hospital, Madison, WI USA; ^5^ Wisconsin Alzheimer's Disease Research Center, Madison, WI USA; ^6^ Wisconsin Alzheimer’s Institute, University of Wisconsin‐Madison School of Medicine and Public Health, Madison, WI USA; ^7^ School of Medicine and Public Health, University of Wisconsin‐Madison, Madison, WI USA; ^8^ Clinical Neurochemistry Laboratory Sahlgrenska University Hospital, Mölndal Sweden; ^9^ Paris Brain Institute, ICM, Pitié‐Salpêtrière Hospital, Sorbonne University, Paris France; ^10^ Institute of Neuroscience and Physiology Sahlgrenska Academy at the University of Gothenburg, Gothenburg Sweden; ^11^ Neurodegenerative Disorder Research Center, Institute on Aging and Brain Disorders, University of Science and Technology of China and First Affiliated Hospital of USTC, Heifei China; ^12^ UCL Institute of Neurology, Queen Square, London United Kingdom; ^13^ Department of Psychiatry and Neurochemistry, Institute of Neuroscience and Physiology, The Sahlgrenska Academy at the University of Gothenburg, Mölndal Sweden; ^14^ Wisconsin Alzheimer’s Disease Research Center, University of Wisconsin School of Medicine and Public Health, Madison, WI USA; ^15^ Department of Psychiatry and Neurochemistry, Institute of Neuroscience and Physiology, The Sahlgrenska Academy, University of Gothenburg, Mölndal, Gothenburg Sweden; ^16^ UK Dementia Research Institute, University College London, London United Kingdom; ^17^ Roche Diagnostics GmbH, Penzberg Germany; ^18^ Hong Kong Center for Neurodegenerative Diseases, Clear Water Bay Hong Kong; ^19^ Wisconsin Alzheimer's Disease Research Center, University of Wisconsin School of Medicine and Public Health, Madison, WI USA; ^20^ Roche Diagnostics International Ltd., Rotkreuz Switzerland; ^21^ University of Wisconsin School of Education, Madison, WI USA

## Abstract

**Background:**

Increasing evidence supports the notion that vascular dysfunction contributes to the evolution of Alzheimer’s disease (AD). Cerebral pulsatility index (PI) is reportedly higher in AD and MCI compared to age matched controls and has been associated with greater beta‐amyloid (Aß) burden. Higher cardiorespiratory fitness (CRF) positively affects vascular function and is associated with lower PI in several large cerebral vessels. Our objective was to examine whether CRF modifies the relationship between PI, Aß burden, and core AD cerebrospinal fluid (CSF) biomarkers.

**Method:**

Cognitively unimpaired adults (n=33, Mean_AGE_=64.0) from the Wisconsin Registry for Alzheimer's Prevention and the Wisconsin Alzheimer's Disease Research Center who met study criteria were included. Aß burden was measured as global cortical PiB‐PET. CSF biomarkers were measured with the NeuroTookKit, a panel of robust prototype assays (Roche Diagnostics International Ltd). PI from bilateral MCA vessels were measured using 4D flow MRI and averaged (PI‐MCA_AVG_). CRF was indexed as VO_2peak_ during a graded exercise treadmill test. A single multivariate regression, (covariate‐adjusted for sex, age, and *APOE*4) examined whether CRF modifies the impact of PI‐MCA_AVG_ on Aß burden and core‐AD CSF biomarkers [phosphorylated Tau 217 (pTau‐217), total Tau (tTau), Aß_42_/Aß_40_, pTau/Aß_42_]. When significant, the PI*CRF interaction term would indicate a differential effect of PI on Aß burden or CSF biomarker as a function of CRF.

**Result:**

There was a significant interaction between PI and VO_2peak_ on **PET Aß burden** (ß = ‐0.05, *p*= 0.04), **Aß42/ Aß40** (ß=0.006, *p*=0.03), **pTau** (ß= ‐3.4, *p*=0.03), **tTau** (ß ‐29.8, p=0.04) and **pTau/Aß42** (ß= ‐0.006, *p*=0.02), indicating less Aß burden and more favorable profiles for all core CSF biomarkers with increasing VO_2peak_ despite elevated PI.

**Conclusion:**

Our findings support the hypothesis that greater CRF modifies the effect of cerebral PI on PET Aß burden and core AD CSF biomarkers, indicating that improved VO_2_ seems to be protective against cerebrovascular alterations known to contribute to AD‐related biomolecular changes.